# RobMedNAS: searching robust neural network architectures for medical image synthesis

**DOI:** 10.1088/2057-1976/ad6e87

**Published:** 2024-08-23

**Authors:** Jinnian Zhang, Weijie Chen, Tanmayee Joshi, Meltem Uyanik, Xiaomin Zhang, Po-Ling Loh, Varun Jog, Richard Bruce, John Garrett, Alan McMillan

**Affiliations:** 1 Electrical and Computer Engineering, University of Wisconsin-Madison, Madison, United States of America; 2 Medical Physics, University of Wisconsin-Madison, Madison, United States of America; 3 Computer Science, University of Wisconsin-Madison, Madison, United States of America; 4 Pure Mathematics and Mathematical Statistics, University of Cambridge, Cambridge, United Kingdom; 5 Radiology, University of Wisconsin-Madison, Madison, United States of America; 6 Biomedical Engineering, University of Wisconsin-Madison, Madison, United States of America

**Keywords:** neural architecture search (NAS), image synthesis, robustness

## Abstract

Investigating U-Net model robustness in medical image synthesis against adversarial perturbations, this study introduces RobMedNAS, a neural architecture search strategy for identifying resilient U-Net configurations. Through retrospective analysis of synthesized CT from MRI data, employing Dice coefficient and mean absolute error metrics across critical anatomical areas, the study evaluates traditional U-Net models and RobMedNAS-optimized models under adversarial attacks. Findings demonstrate RobMedNAS’s efficacy in enhancing U-Net resilience without compromising on accuracy, proposing a novel pathway for robust medical image processing.

## 1. Introduction

Medical image synthesis plays a significant role in modern healthcare. Such tools are used in medical image reconstruction and other applications [1]. In this work we study the application of synthesis in a MRI to CT conversion task. In this way, MR images alone can be used to process radiotherapy treatment simulations and/or combined PET/MR reconstruction without the need to acquire a CT [2–6] Historically, various U-Net architectures have been proposed and show high accuracy [7, 8]. For example, a U-Net structure with modified Inception V3 [9] blocks was designed in [7].

Although more progress has been made in improving the accuracy of U-Nets, only a few studies were focused on the robustness over adversarial examples that may be utilized for fraudulent purposes [10]. In [11], experimental results on skin lesion classification and whole-brain segmentation showed that U-Net [12] are vulnerable to adversarial attacks based on the fast gradient sign method (FGSM) [13]. Moreover, a systematic study [14] is conducted on the robustness of the classic 2D U-Net [12] and the 3D U-Net model [15] (winner of the MICCAI Brain Tumor Segmentation (BraTS) 2017 challenge [16]). Both U-Net architectures suffer from up to 65% loss in the Dice coefficient under FGSM. This work also explored the effectiveness of two popular defense methods: distillation [17] and adversarial training [13]. The traditional adversarial training method can successfully improve the robustness of U-Nets, but accuracy on clean data drops unless rectifications are applied, as discussed in existing literature [18]. Despite these prior efforts, the robustness of U-Net architectures for medical image synthesis has not been fully studied and there remains an unmet need to better understand the performance limitations of these networks as they become more widely used.

A recent study [19] shows that robustness appears to be an intrinsic property of a network’s architecture for image classification, and several weight sharing based neural architecture search (NAS) methods have been proposed [20] to identify robust architectures. Another important line of NAS research is based on Bayesian optimization. The core idea is to establish a probability model for either the likelihood or posterior between the architectural space and metric space, such as Gaussian processes and the Tree-structured Parzen Estimator (TPE) approach. The parameters of the probability model are estimated by using previously explored data, and the most promising architecture is identified according to a criterion defined on the probability model, e.g., expected improvement (EI). The premise of this existing work is that specific network architectures are inherently more robust, and there exists distinct benefits to identifying a network that is naturally resilient to worst-case adversarial attacks versus one that may have unknown or poor resilience.

In this paper, we explore the robustness of U-Nets in the context of medical image synthesis and explore the effectiveness of adversarial training. We then propose RobMedNAS, a neural architecture search method that applies Bayesian optimization to automatically search robust U-Net architectures. Experiment results demonstrate that the RobMedNAS identified U-Net is more robust and equivalently performant when compared to a handcrafted U-Net given.

## 2. Materials and methods

Data were collected from 79 patients at our institution who had undergone a clinical CT and MRI scan on the same day as described in [7]. This study was approved by the Institutional Review Board (IRB) of the University of Wisconsin-Madison (IRB protocol 2016–0418). All research was conducted in accordance with the principles of the Declaration of Helsinki. For image preprocessing, we applied volume-wise z-score normalization for each subject to the MR images. For the CT images, we first added a bias of −1000, and then utilized normalization with fixed mean and deviation of 1250 and 2500, respectively. Finally, we converted each volume into 182 axial 2D slices, with the dimension of each slice 256 × 256.

We held out 7 subjects as the test dataset, consisting of 1274 2D images. The rest subjects were randomly split into training and validation datasets, with 63 and 9 subjects, respectively. Namely, the training dataset contains 11466 images and the validation dataset has 1638 images. For RobMedNAS, we selected 6 subjects from the training dataset and form the subdataset, consisting of training, validation, and test data by the ratio of 4:1:1.

### 2.1. FGSM attack

To assess the robustness of a trained model, we utilized the first-order attack FGSM that constructs perturbations abased on the gradient of a loss function evaluated on input images for a given trained network. Specifically, FGSM perturbs an image $S$ according to the equation:

\begin{eqnarray*}{S}_{adv}=S+\varepsilon \cdot {\mathrm{sign}}\left({{\mathrm{\nabla }}}_{S}l\left(S,T\right)\right),\end{eqnarray*}

where $\varepsilon \geqslant 0$ is the magnitude of perturbation on individual pixels, and $l\left(\cdot \right)$ is the loss function with respect to the input image $S$ and its corresponding ground truth label $T.$ The perturbed image ${S}_{adv}$ was fed into the trained model. We defined the performance of the trained model on the perturbed images as the robustness. Meanwhile, the performance on the clean images $S$ was defined as accuracy.

### 2.2. RobMedNAS

**Figure 1.**
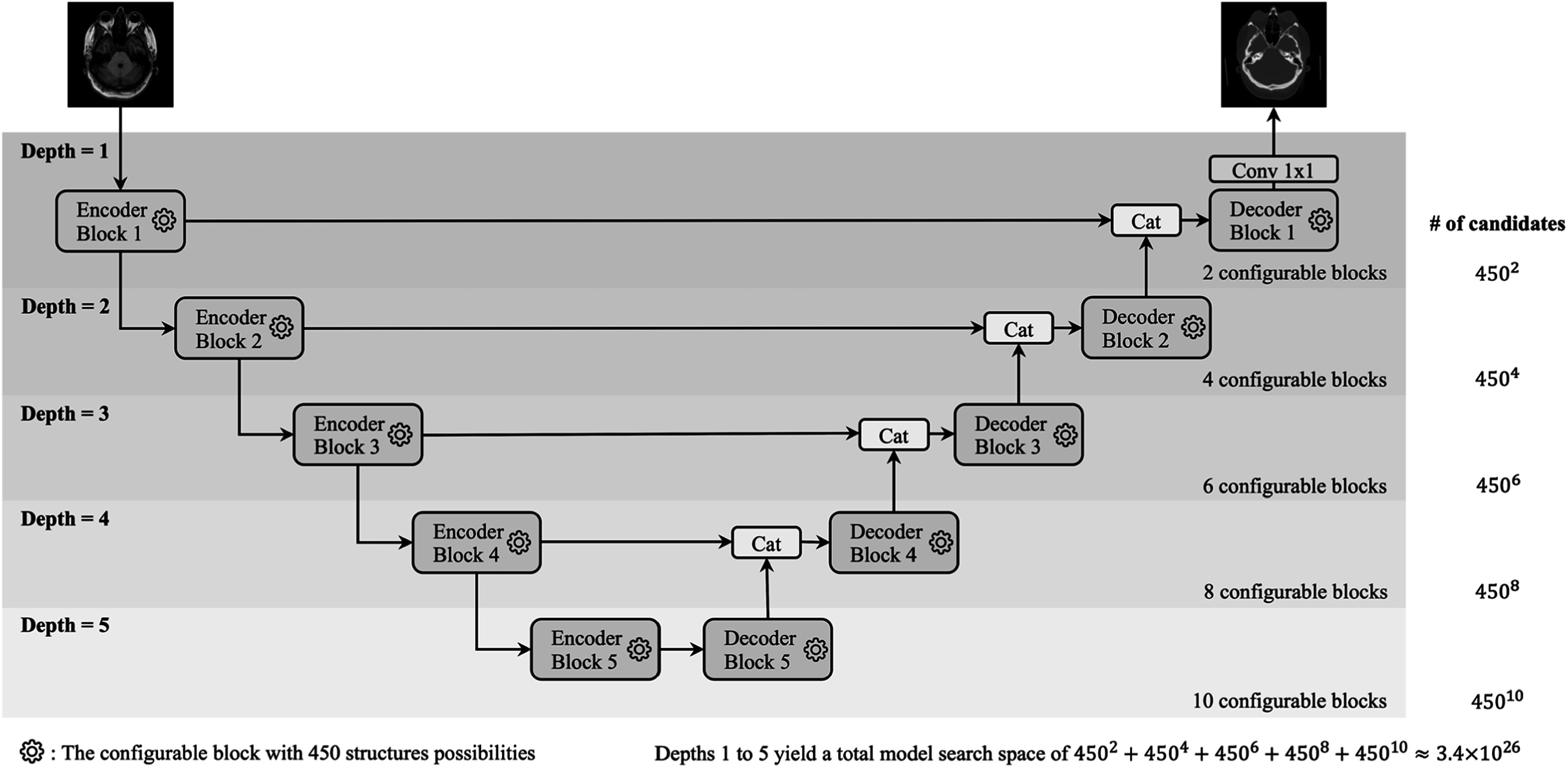
This figure illustrates the generalized U-Net architecture. The depth of the U-Net structure can be selected from 1 to 5, indicating configurations ranging from one encoder and one decoder to five encoders and five decoders. Each encoder and decoder can be configured independently. As shown in figure 2 and detailed in table 1, there are 450 possible configurations for each encoder and decoder. The number of candidate models for each depth is indicated on the right-hand side, and the total search space, computed at the bottom right, is approximately.4 × 10^26^.

**Figure 2.**
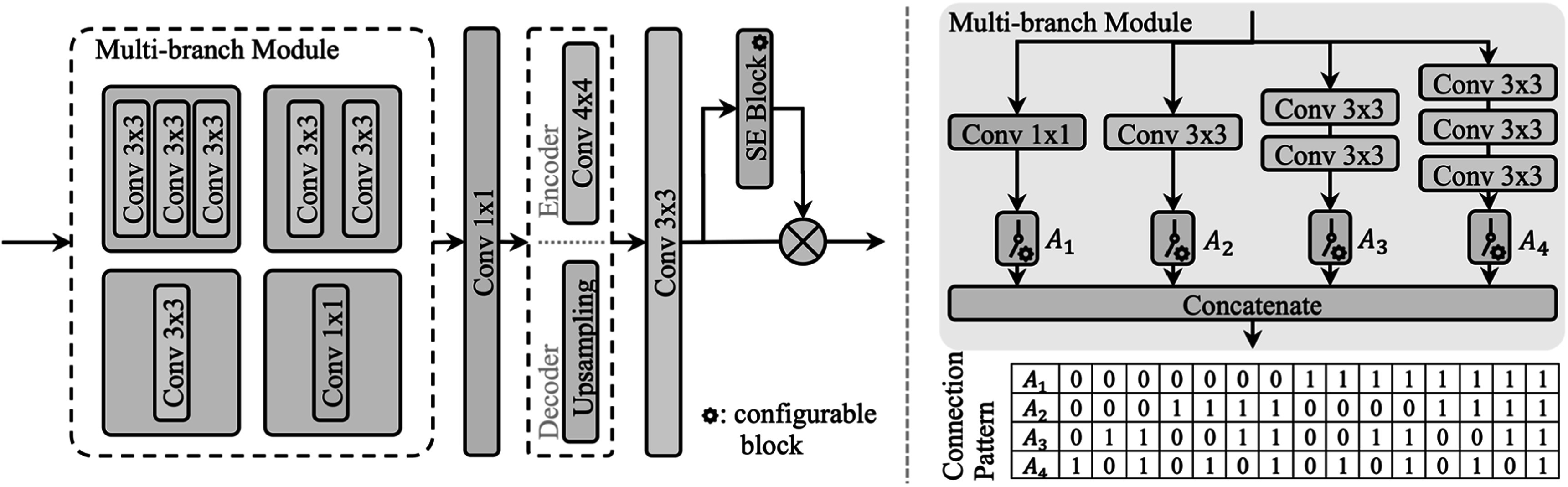
This figure depicts the structure of the configurable block. There are options for A_1_, A_2_, A_3_, and A_4_ to be turned on or off, representing different combinations of convolutional kernels. Since at least one convolutional layer is required, there are 2^4^−1 = 15 possible configurations. Additionally, the number of convolution filters can be selected, though this is not shown in the figure. The figure also allows for the inclusion of SE (Squeeze-and-Excitation) Blocks and the adjustment of their reduction ratios.

We proposed RobMedNAS to obtain robust architectures, which consists of three components: search space, search strategy and performance estimation. The search space was created by generalizing the 2D U-Net [7] architecture for synthesis, as visualized in figure 1. There are a maximum of five configurable blocks for each encoder and decoder, and the depth of the architecture is controlled by configurable switches. In figure 2, each configurable block has a multi-branch module, a point-wise convolutional layer, a upsampling or a convolutional layer with kernel size 4 and stride 2 for downsampling depending on if it is in the encoder or decoder block, a convolutional layer with kernel size 3 and a Squeeze-and-Excitation (SE) block [21]. In the multi-branch module, there are four independent branches controlled by configurable switches, denoted by ${A}_{1},$ ${A}_{2},$ ${A}_{3}$ and ${A}_{4},$ respectively. Because at least one branch should exist in the module, there are in total 15 connection patterns ranging from 0001 to 1111, where 0 represents the corresponding branch is abandoned. We listed all hyperparameters and their possible values in table 1. Consequently, we obtained 450 different structures for each encoder or decoder block, and our search space contains around $3.4\times {10}^{26}$ potential U-Net architectures.

**Table 1.** Description of the defined search space in RobMedNAS. There are 450 candidate structures for each block. It consists of around 3.4 × 10^26^ different potential U-Net architectures.

Hyperparameters for the configurable block	Candidate values
Connection Pattern	{1, 2, 3, 4, 5, 6, 7, 8, 9, 10, 11, 12, 13, 14, 15}
Number of filters in Convolutional Layers	{8, 16, 32, 64, 128, 256}
SE Block [21]	{True, False}
Reduction Ratio of the SE Block	{2, 4, 8, 16}
Number of Candidate Blocks	$15\times 6\times \left(1+4\right)=450$
Total Size of the Search Space	${450}^{4}{+450}^{6}{+450}^{8}{+450}^{10}\approx 3{.4\times 10}^{26}$

Due to the large search space, it is challenging to train and evaluate all architectures to identify the most robust architecture. Therefore, we used a subdataset to estimate the robustness of each architecture, expressed as

\begin{eqnarray*}\mathop{\max }\limits_{x\in {\mathscr{X}}}\displaystyle \frac{1}{M}\displaystyle \displaystyle \sum _{\left(S,T\right)\in {\hat{D}}_{test}}f\left(t\left({S}_{adv};{\theta }_{x}^{\ast }\right),T\right),\end{eqnarray*}

where $f$ is the metric function to measure the closeness of the model prediction $t\left({S}_{adv};{\theta }_{x}^{\ast }\right)$ and the ground truth label $T,$ such as Dice coefficients and MAE. We saved model weights after each epoch of the training process on the training data ${\hat{D}}_{train}$ in the subdataset, and ${\theta }_{x}^{\ast }$ is the optimal weights of the architecture $x$ chosen from the search space ${\mathscr{X}},$ which minimize the loss on the validation data ${\hat{D}}_{val}$ in the subdataset. ${\hat{D}}_{test}$ is the test data in the subdataset with $M$ images.

However, it is still difficult to explore all architectures in the search space. A simple solution is to randomly select $N$

architectures in the search space and identify the one with best robustness on the test dataset. We called this method RobMedNAS-R. Moreover, we applied a Tree-structured Parzen Estimator (TPE) [22], which is a Bayesian Optimization [23] method, to improve search efficiency and call this method RobMedNAS-T. Details of TPE were discussed in the Supplementary Materials. Because RobMedNAS-T is able to utilize the previous explorations to avoid bad architectures and move towards the best architecture in each iteration, it is expected to identify a more robust architecture than RobMedNAS-R.

In this study, we present several U-Net models for comparison: the human-crafted U-Net model without adversarial training (‘Baseline’), the human-crafted U-Net model with adversarial training (‘Baseline w/AT’), the randomly structured U-Net (‘Random’), the best model selected from the search budget $N$ through random choice (‘RobMedNAS-R’), and the best model selected using the Tree-structured Parzen Estimator (TPE) method within the same search budget (‘RobMedNAS-T’).

In our experiments, the search budget $N$ of RobMedNAS was set to 200. In each iteration, the candidate architecture was trained 10 epochs using a batch size 6 on the training data in the subdataset. We used the ADAM [24] optimizer with AMSgrad [25] and a learning rate of 1e-4. The loss function is MSE (Mean Square Error), and the magnitude of FGSM $\varepsilon $ is 0.2 when evaluating the robustness on the validation and test data in the subdataset. We repeated the RobMedNAS-R and RobMedNAS-T method 15 times, respectively. Finally, for each method, we obtained 15 robust architectures, and trained them on the full training dataset using the regular training strategy to identify the one with the best robustness on the test dataset. We randomly chose 15 different architectures from the search space and choose the most robust one for fair comparison. The selection of 15 robustness architectures is based on statistical testing, ensuring that this number is sufficient to demonstrate the significant difference between the results obtained from our search strategies.

For the purpose of our evaluation, we defined robustness of a model as the Dice coefficient or MAE between its predictions on the perturbed MRI images using FGSM and the ground truth CT images.

### 2.3. Regular training strategy

We trained all architectures including the handcrafted architecture [based on [7]] on the full training dataset by directly inheriting hyperparameters from [7]. Specifically, all models were trained 150 epochs, with batch size of 6, ADAM as the optimizer with AMSgrad and a learning rate of 1e-4. We adopted an early stopping strategy with a patience of 5 epochs using the validation dataset.

### 2.4. Adversarial training

For the handcrafted architecture, we utilized the adversarial training [13] method for robustification. It has been successfully applied to robustifying U-Nets for medical image segmentation [7]. In particular, we use the adversarial objective function based on FGSM as an effective regularizer:

\begin{eqnarray*}\tilde{l}\left(S,T\right)=\beta l\left(S,T\right)+\left(1-\beta \right)l\left({S}_{adv},T\right),\end{eqnarray*}

where $\beta \sim {\mathrm{Bernoulli}}\left({p}_{r}\right)$ is a binary Bernoulli random variable and ${p}_{r}$ is the probability of replacement. For a batch of samples with size $B,$ the total loss is then calculated as

\begin{eqnarray*}\begin{array}{c}\tilde{L}=\displaystyle \frac{1}{B}\displaystyle \sum _{i=1}^{B}\tilde{l}\left({S}_{i},{T}_{i}\right)\\ =\,\displaystyle \frac{1}{B}\displaystyle \sum _{i=1}^{B}{\beta }_{i}l\left({S}_{i},{T}_{i}\right)+\left(1-{\beta }_{i}\right)l\left({S}_{adv}^{i},{T}_{i}\right),\end{array}\end{eqnarray*}

where ${\beta }_{i}$ s are independent Bernoulli random variables with identical distribution. In our experiments, we set the probability of replacement ${p}_{r}$ as 0.5 and the magnitude of adversarial noise $\varepsilon =0.2.$

### 2.5. Metrics and statistical analysis

We considered two metrics (Dice coefficient and MAE) between the predictions of trained models and the ground truth CT images in four regions: Whole Brain, Air (HU < 500), Bone (HU > 500) and Soft Tissue (−500 < HU < 300). It is notable that all metric values are calculated over the whole 3D volume of each subject in the test dataset.

Given a trained model, we utilized the FGSM attack to generate adversarially perturbed test data. The accuracy and robustness of the model are then evaluated by using the above two metrics on the original clean test dataset and the adversarially perturbed test data, respectively. Wilcoxon signed-rank tests are used to determine whether one architecture has better accuracy or robustness than another with statistical significance. The p-values are adjusted using the Bonferroni Correction, a multiple comparison correction that helps control the familywise error rate.

### 2.6. Study on the effect of weight initialization and training data on robustness

Given the best robust architecture identified by RobMedNAS-T, we first studied the effect of weight initialization. In particular, we randomly initialized the model weights with 11 different random seeds and adopted the regular training strategy as described above. The robustness of trained models was evaluated on the test data. Then we studied the effect of training data on the robustness using a cross validation strategy with a fixed random seed. Specifically, we first split the training dataset into eight groups uniformly, and then reserved one group as the validation dataset and used the rest groups as the training dataset. The validation dataset was used for early stopping. After training, all eight models were evaluated on the test data.

### 2.7. Progressive corruptions

To evaluate the robustness of different models under varying levels of corruption, we tested the models using datasets subjected to progressively increasing adversarial attacks (FGSM) and Gaussian noise. The FGSM attack was parameterized by epsilon values ranging from 0.02 to 0.2, with increments of 0.02. For Gaussian noise, which was added to the input data normalized to zero mean, we varied the standard deviation from 0.025 to 0.25 in increments of 0.025. Performance was measured using Mean Absolute Error (MAE) and Dice Coefficients, averaged across different regions including brain, air, bone, and soft tissues.

## 3. Results

### 3.1. Performance of RobMedNAS.

The Dice coefficients and MAE performance of robust architectures identified by our proposed RobMedNAS method with and without adversarial noise are listed in tables 2 and 3, respectively. We observe that standard adversarial training can successfully improve the robustness of deep learning models, but the accuracy on the clean data drops. For example, the handcrafted model [7] after adversarial training can achieve an increase of the Dice coefficients on the bone and the soft tissue region by around 0.2 and 0.06 with the existence of adversarial noise, respectively. However, on clean data, the Dice coefficients dropped by nearly 0.01 in both regions. This tradeoff between accuracy and robustness becomes more obvious on the soft region under the metric of MAE. Therefore, it may be desirable to optimize the model architecture for better robustness to avoid the tradeoff and training inefficiency [26] brought by adversarial training.

**Table 2.** The Dice coefficient values of different models on the region of brain, air, bone and soft tissues. We consider the human designed U-Net [7] with and without Adversarial Training (AT) as the baselines. We repeated the random and our proposed RobMedNAS methods 15 times and report the models with the best robustness (FGSM with $\varepsilon =0.06$) on the Bone region. All models are evaluated on the test dataset w/o and w/adversarial noise (FGSM with $\varepsilon =0.06$), shown in the column of ‘Nrm.’ for normal accuracy, ‘Rob.’ for robust accuracy, respectively. The best performance is shown in bold. Values with a star (*) indicate statistically significant differences to the baseline without AT after Bonferroni correction $\left(n=3\right)$.

Model	Brain		Air		Bone		Soft	
	Nrm.	Rob.	Nrm.	Rob.	Nrm.	Rob.	Nrm.	Rob.
Baseline	0.970	0.919	0.986	0.959	0.786	0.539	**0.921**	0.839
	± 0.005	± 0.033	± 0.002	± 0.021	± 0.065	± 0.105	± 0.015	± 0.031
Baseline w/AT	0.970	**0.968**	0.986	**0.985**	0.775	**0.742**	0.908	0.900
	± 0.004	± 0.004	± 0.002	± 0.002	± 0.051	± 0.060	± 0.015	± 0.016
Random	0.968	0.959*	0.985	0.981*	0.754	0.651*	0.901	0.875*
	± 0.005	± 0.010	± 0.003	± 0.005	± 0.068	± 0.087	± 0.014	± 0.020
RobMedNAS-R	0.971*	0.960*	0.987*	0.981*	0.801*	0.678*	0.920	0.884*
	± 0.005	± 0.009	± 0.002	± 0.005	± 0.059	± 0.084	± 0.014	± 0.022
RobMedNAS-T	0.970	0.965*	0.986	0.984*	0.782	0.712*	0.920	0.905*
	± 0.004	± 0.006	± 0.002	± 0.003	± 0.073	± 0.093	± 0.014	± 0.017

**Table 3.** The mean absolute values (MAE) of different models on the region of brain, air, bone and soft tissues. We consider a human designed U-Net as baseline with and without Adversarial Training (AT) as the baselines. We repeat the random and our proposed RobMedNAS methods 15 times and report the models with the best robustness (FGSM with $\varepsilon =0.06$) on the Bone region. All models are evaluated on the test dataset w/o and w/adversarial noise (FGSM with $\varepsilon =0.06$), shown in the column of ‘Nrm.’ for normal accuracy and ‘Rob.’ for robust accuracy, respectively. The best performance is shown in bold. Values with a star (*) indicate statistically significant differences to baseline without AT after Bonferroni correction $\left(n=3\right)$.

Model	Brain		Air		Bone		Soft	
	Nrm.	Rob.	Nrm.	Rob.	Nrm	Rob.	Nrm.	Rob.
Baseline	**44** ± 6	75 ± 16	41 ± 4	104 ± 28	27 ± 4	50 ± 10	**21** ± 3	29 ± 5
Baseline w/AT	51 ± 6	54 ± 6	**37** ± 2	**38** ± 3	29 ± 3	**32** ± 4	25 ± 3	26 ± 3
Random	53 ± 5	63* ± 7	44 ± 5	66* ± 10	30 ± 4	40* ± 5	26 ± 2	28 ± 3
RobMedNAS-R	47 ± 6	63* ± 7	41 ± 3	68* ± 9	26* ± 4	37* ± 5	25 ± 2	31 ± 3
RobMedNAS-T	**44** ± 6	51* ± 7	43 ± 3	52* ± 6	27 ± 5	33* ± 5	22 ± 2	24* ± 3

We randomly chose 15 different architectures in the search space and found that the best robust architecture still has a large gap compared to the handcrafted model with adversarial training, i.e., Dice coefficients of 0.02 on clean data (accuracy) and 0.09 on adversarially perturbed data (robustness) in the bone region. This indicates that there are poorly-performing architectures in the search space.

The proposed RobMedNAS method can identify the architecture with both good accuracy and robustness. Considering Dice coefficient as the metric, the architecture identified by RobMedNAS-R achieves the best accuracy on the region of Brain, Air and Bone; however its robustness is less than the handcrafted model with adversarial training, especially on the bone region. However, the RobMedNAS-T identified architecture can achieve better accuracy and comparable robustness compared to the handcrafted model with adversarial training. Namely, when evaluated on clean data (accuracy), its Dice coefficients on the bone region is 0.03 higher, whereas it was only 0.03 less in Dice coefficients on the bone region but even 0.01 higher on the soft region when evaluated on adversarially perturbed data (robustness). It is notable that adversarial training brings high computational cost, because it has an extra forward and backward pass for each training iteration, and it requires more training iterations to converge. The structure of network is described in table 4.

**Table 4.** The best robust architecture identified by RobMedNAS-T.

Hyperparameters	Enc1	Enc2	Enc3	Enc4	Enc5	Dec1	Dec2	Dec3	Dec4	Dec5
Connection Pattern	1110	0110	0111	1110	0010	0111	0001	1100	0010	0010
Number of Filters	128	256	64	128	256	8	128	64	128	8
SE Block	True	True	False	True	False	True	True	True	False	True
Reduction Ratio	16	4	—	8	—	8	8	8	—	4

### 3.2. Visualization of model predictions w/and w/o adversarial noises

**Figure 3.**
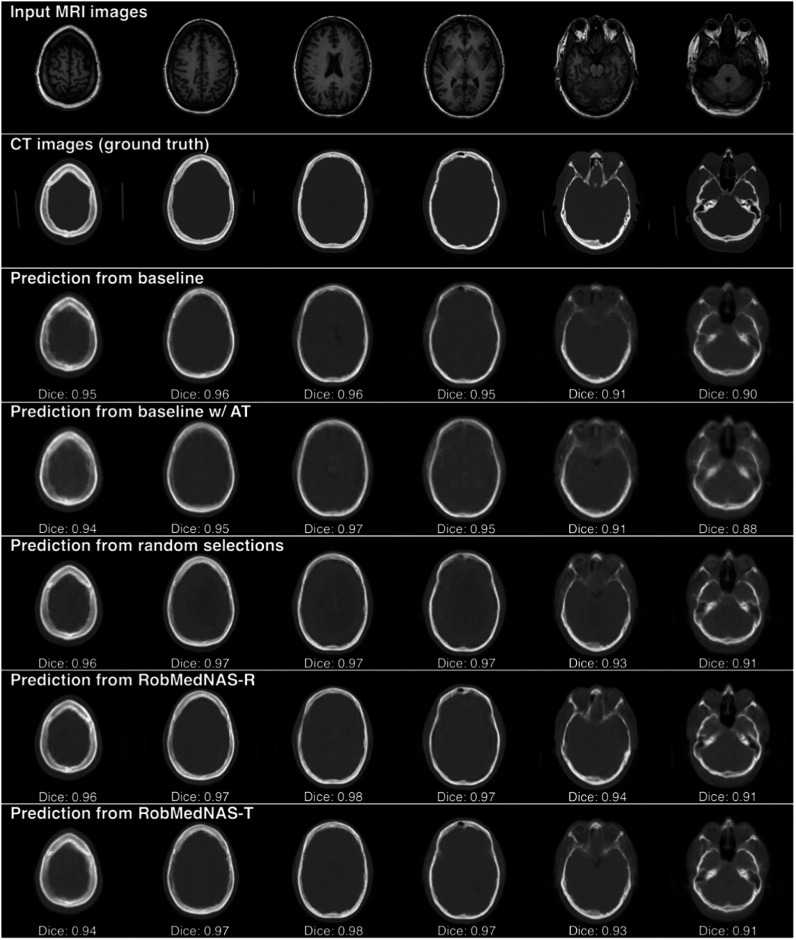
The performance of different models on selected MRI slices. We calculate the Dice coefficient values by averaging on the region of brain, air, bone and soft tissues.

We visualized the predictions of different models on selected MRI slices. In figure 3, we observe that all models can output synthetic images close to the ground truth CT images given clean input MRI images. We calculate the Dice coefficients for region of brain, air, bone and soft tissuesbetween the prediction and ground truth, and show the average value in each image. Even for the most challenging sample (the fifth column), all models can still achieve at least 0.7 average Dice coefficient over all 4 regions. Moreover, the RobMedNAS identified models in the last two rows can generate more details in the predictions, especially in the fourth column, showing that they are more accurate than the other architectures.

**Figure 4.**
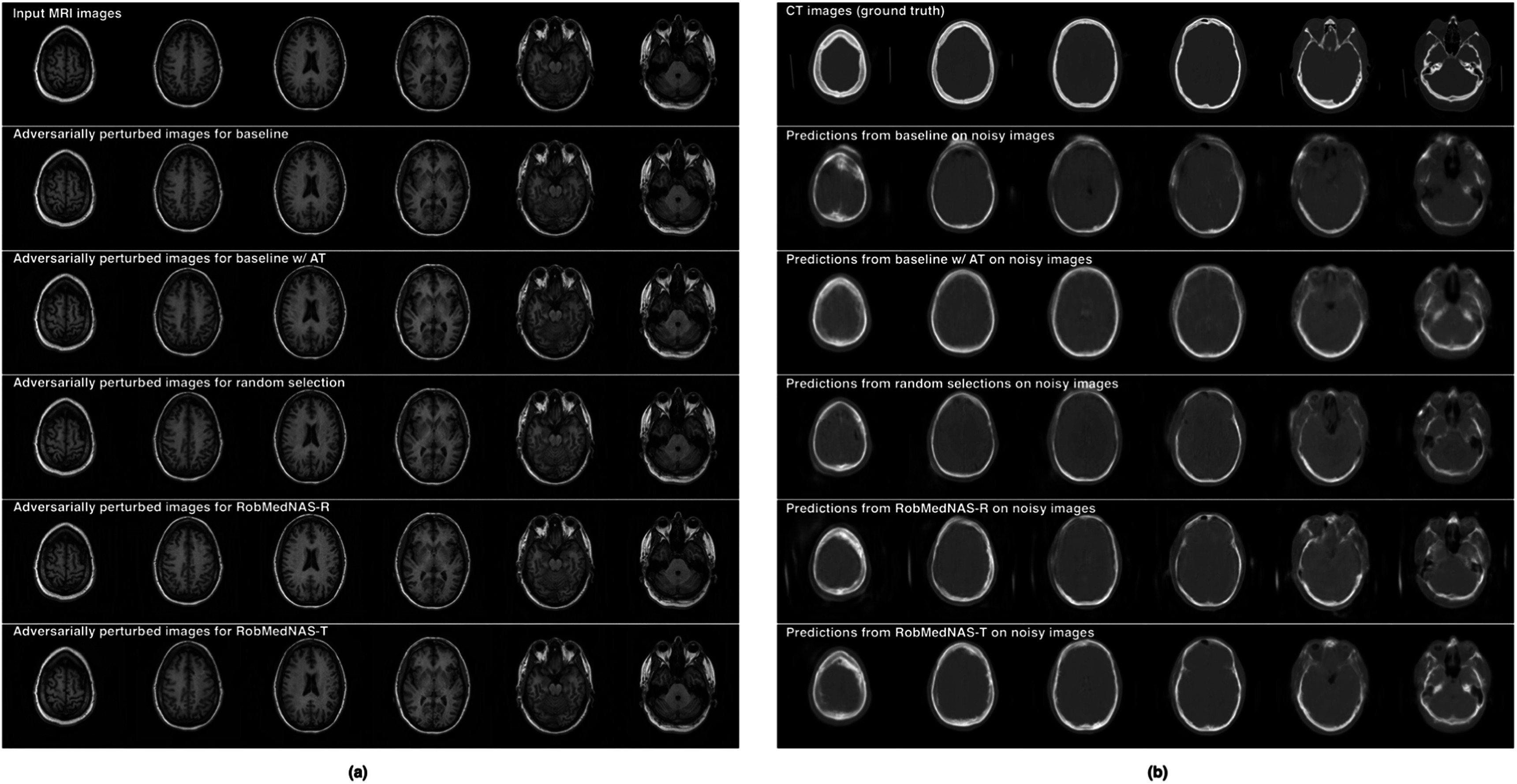
(a) The visualization of adversarially perturbed images by using FGSM attack with $\varepsilon =0.15.$ (b) The performance of different models on the perturbed images. We calculate the Dice coefficient values by averaging the region of brain, air, bone and soft tissues.

We compared the robustness of all models in figure 4. Given the trained models and clean input MRI images, we generate adversarial noise by using FGSM with $\varepsilon =0.15,$ and show the perturbed images for each model in figure 4(a), The adversarial noise is too subtle to be recognized by our human eyes. However, the predictions of most models are largely affected as shown in figure 4(b), except the RobMedNAS-T identified architecture and the model with adversarial training.

### 3.3. Statistical comparisons

**Figure 5.**
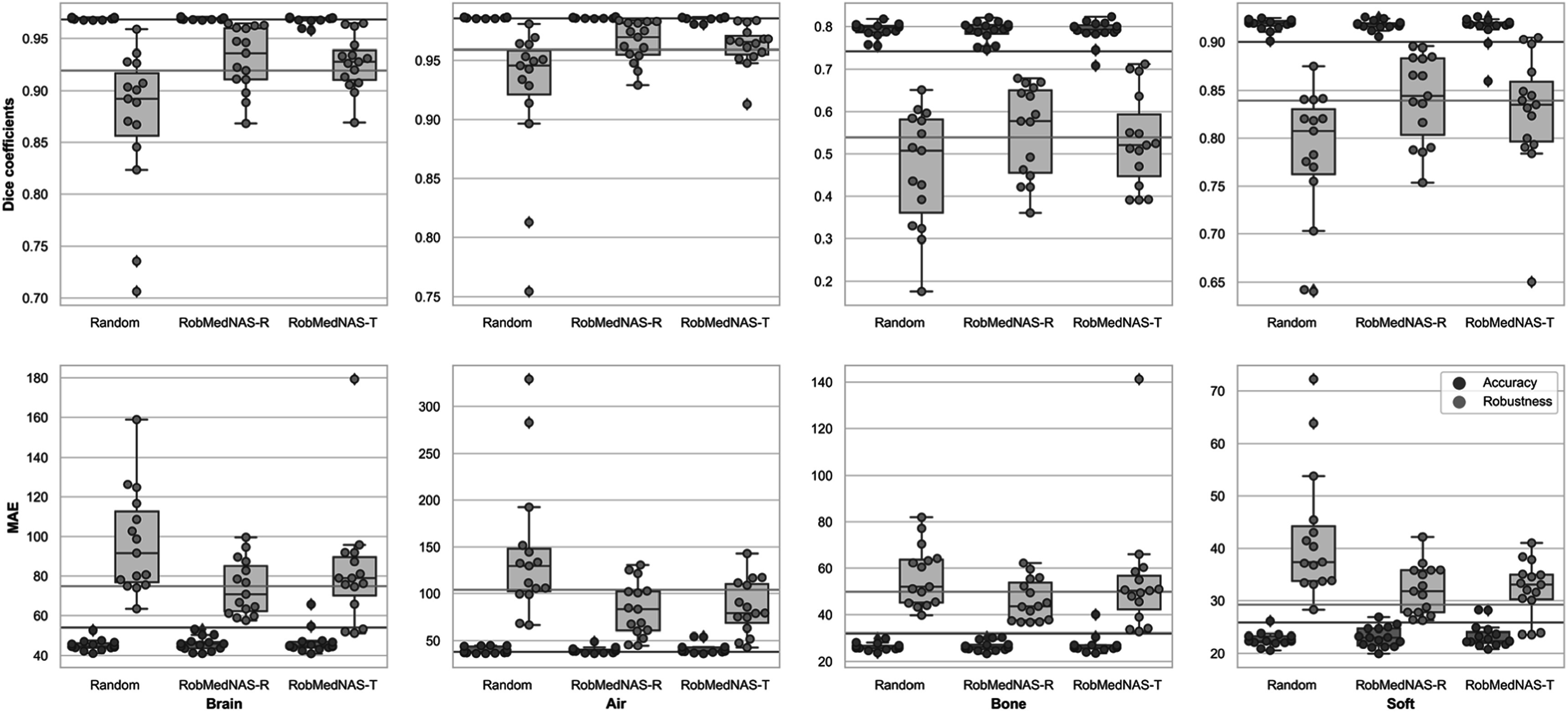
The performance of the architecture identified by different methods on clean data (accuracy) and adversarially perturbed data (robustness). We ran all three methods 15 times. The blue and red line represent the robustness of the baseline model with and without adversarial training, respectively. Comparing blue boxes in each subplot, architectures generated by different methods achieve similar accuracy over all four regions: Whole Brain, Air, Bone and Soft Tissue. Additionally, each orange box shows worse performance than its corresponding blue box, indicating that the existence of adversarial perturbations deteriorates the performance of all architectures. However, more than half of architectures identified by RobMedNAS achieves better performance than the baseline model without adversarial training (orange line). Some architectures identified by RobMedNAS can even outperform the baseline model with adversarial training (blue line).

We evaluated both the accuracy and robustness of all architectures identified by different methods in 15 independent runs on the test dataset. In figure 5, all architectures showed good accuracy in both Dice coefficients and MAE. Considering the Dice coefficients on the bone region, the percentage of architectures that were higher than 0.8 is 33.3%, 40.0% and 40.0% for the random, RobMedNAS-R and RobMedNAS-T method, respectively.

However, these architectures have varying robustness. Most architectures are vulnerable to adversarial noise and suffer from a performance degradation, especially on the region of bone and soft tissues. However, the RobMedNAS-identified architectures are more robust to the random method. We show the robustness of the handcrafted model w/o and w/adversarial training by red and blue lines, respectively. More than 50% of architectures identified by RobMedNAS-R are more robust than the handcrafted model with respect to the Dice coefficients of the bone region. It is notable that although in average RobMedNAS-R can identify more robust architecture than RobMedNAS-T, the architectures identified by RobMedNAS-T are more likely to yield the highest robustness. For example, the best RobMedNAS-T identified architecture can outperform the model with adversarial training in both metrics on the region of soft tissues. Moreover, when comparing the worst-performing architectures identified by different methods, RobMedNAS-T still achieved the best Dice coefficients on adversarially perturbed data, i.e. robustness.

### 3.4. The effect of training data and weight initialization on robustness of the best architecture

**Figure 6.**
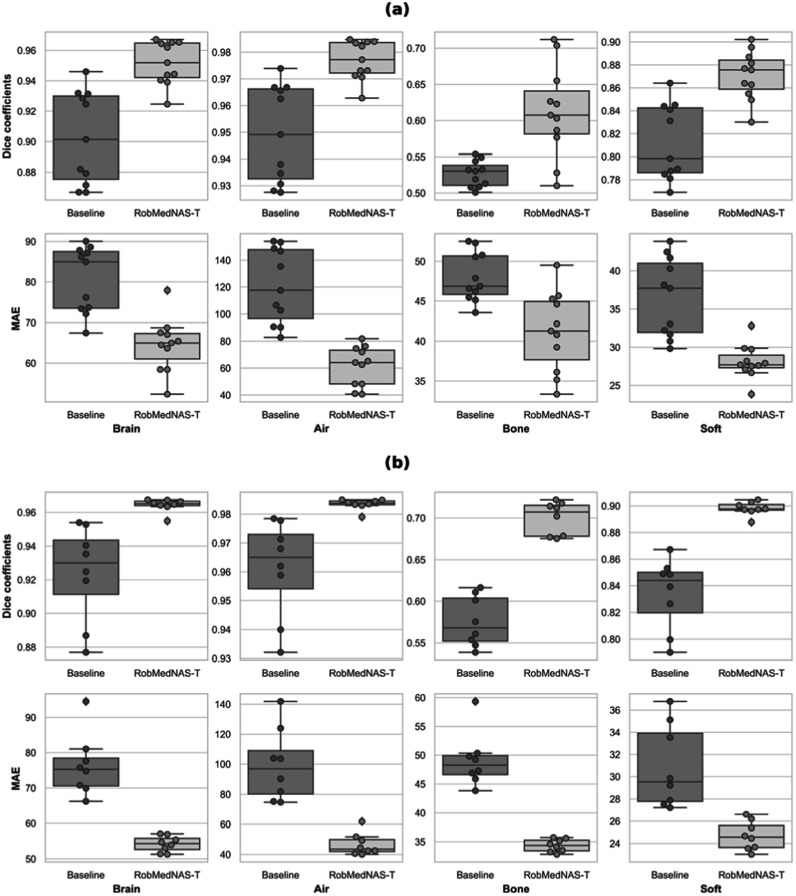
(a) The robustness of the best architecture with different random initialization of weights. Overall, the RobMedNAS-T architecture has improved performance with higher Dice coefficients and lower MAE compared to the baseline architecture. (b) The robustness of the best architecture with different training data. In general, the RobMedNAS-T architecture has improved performance with higher Dice coefficients and lower MAE compared to the baseline architecture.

In figure 6(a), we observe that weight initialization is critical to overall model performance. However, the RobMedNAS-T identified architecture still had higher robustness than the handcrafted model on average. Moreover, it had a smaller variance in robustness in most metrics except the Dice coefficients and MAE on the bone region, indicating better stability despite random weight initialization. The Dice coefficients and MAE values on adversarially perturbed data (robustness) of models trained on different data is shown in figure 6(b), Compared to the handcrafted model, the robust architecture identified by RobMedNAS-T were less sensitive to training data, and consistently performed better in all metrics.

### 3.5. Model performance under progressive corruptions

**Figure 7.**
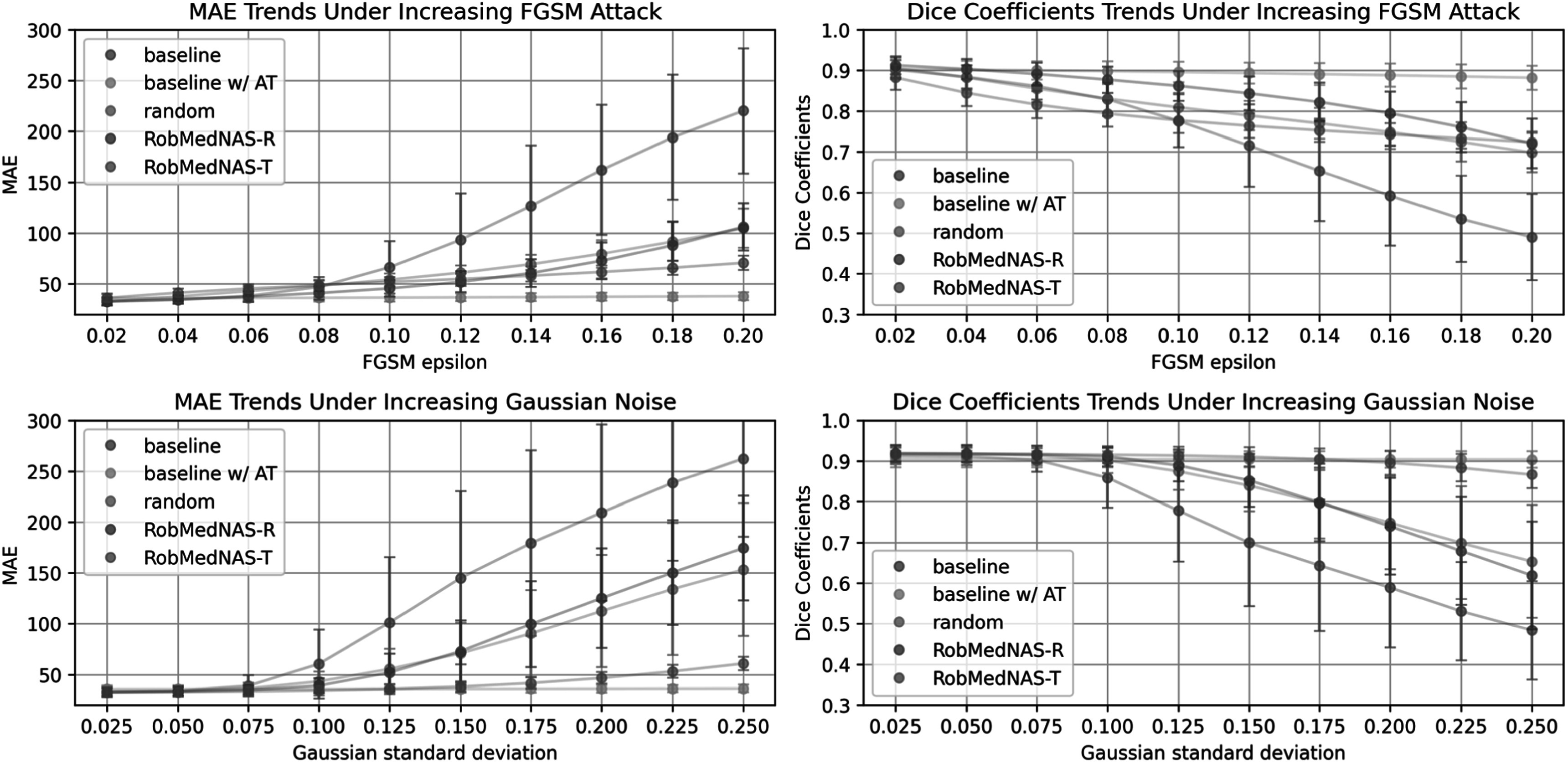
The upper row depicts the FGSM attack results, while the lower row shows the Gaussian noise results. The left column illustrates the Mean Absolute Error (MAE), and the right column presents the Dice Coefficients. Metrics are averaged across the regions of the brain, air, bone, and soft tissue. Error bars represent the standard deviation from the testing samples.

In figure 7, we present the performance of the models under increasing levels of FGSM attacks and Gaussian noise. For the FGSM attack, we observed a significant drop in performance for the baseline model as the epsilon parameter increased. In contrast, the baseline model with adversarial training (Baseline w/AT) maintained consistently high performance, demonstrating that adversarial training is an effective strategy for mitigating FGSM attacks. However, it is important to note that adversarial training represents the upper limit of robustness and leads to a relative performance drop in clean datasets in tables 2 and 3. Moreover, the RobMedNAS models, which are identified using our proposed search strategy without adversarial training, exhibited robust performance against FGSM attacks while also achieving superior results on the clean dataset. This highlights the effectiveness of the RobMedNAS models in maintaining robustness through intrinsic structural properties rather than adversarial training.

Regarding Gaussian noise, the RobMedNAS-T model demonstrated robust performance even when subjected to high standard deviations of noise, indicating its resilience to this type of corruption. Overall, the RobMedNAS models show that it is possible to achieve significant robustness against both adversarial attacks and input noise solely through architectural design, without the need for adversarial training.

## 4. Discussion

We have demonstrated that handcrafted U-Net architectures can be sensitive to adversarial perturbations in an MR-to-CT synthesis application, and that adversarial training can improve the robustness by sacrificing the accuracy on the clean data. Experimental results imply the existence of robust architectures can retain good performance under adversarial attacks. Since robustness has become a property of these architectures, robustification methods such as adversarial training are not required, which typically have high computational complexity and introduce the tradeoff between accuracy and robustness. Furthermore, we have developed an efficient algorithm RobMedNAS to identify robust architectures. On the dataset collected from our own institution, the RobMedNAS-identified architecture had better accuracy and robustness than the handcrafted architecture. Moreover, it can still achieve good robustness on different training data with various weight initialization.

In this work, we have mainly focused on the method for efficiently identifying robust architectures. It is interesting to obtain more architectures by using RobMedNAS and analyze the statistics, which is likely to guide the future efficient architecture design. Furthermore, recent work [27–31] shows that differentiable architecture search utilizes the continuous relaxation of the architecture representation, allowing efficient search of the architecture using gradient descent. We plan to investigate whether differential architecture search can identify robust architectures for medical imaging. Lastly, traditional robustification methods, such as adversarial training, are potentially data specific. Namely, they likely need to be applied to improve robustness on each dataset. However, since robustness has been one property of the architecture identified by our proposed RobMedNAS, we expect it can still be robust to adversarial attacks on other datasets, which constitutes part of our future work. Additionally, we plan to conduct a more detailed analysis of the architectural differences in the future. By doing so, we aim to strengthen our results and gain a deeper understanding of the factors contributing to improved robustness. This will enhance the comprehensiveness and applicability of our findings.

This work has several limitations. First, we have focused on a single class of 2D U-Nets for medical image synthesis. It would be interesting to see if our proposed method can efficiently identify 3D U-Net architectures, which may yield improved results, but are computationally more complex. Moreover, other architectures such as vision transformers have shown great potential in many computer vision domain applications. Recently, ResViT [32] successfully combined transformer blocks and convolutional layers to achieve good performance in medical image synthesis. Studies have suggested that transformers can exhibit better robustness than CNNs [33–35]. Therefore, further study of the robustness of transformer-based architectures using the RobMedNAS-T concept is warranted. Second, we have mainly studied adversarial attacks based on FGSM, since they generate adversarial perturbations in a fast and simple way. However, one could similarly adopt other attack methods such as PGD [36], Deepfool [37], and AutoAttack [38]. These methods could lead to more effective attacks. Third, we only consider MR-to-CT tasks. It is still remains to be studied whether robust architectures also exist for classification and segmentation. Therefore, more work is needed to extend the RobMedNAS method to other tasks to verify its generalizability. Fourth, we only consider the most standard method, adversarial training, to robustify models as a baseline. Recent literature investigates its side effect of decreasing accuracy, and proposes several rectification methods for retaining accuracy when improving robustness of [18]. However, these methods do not take computational complexity into account, and conversely bring extra computations to balance accuracy and robustness.

## 5. Conclusion

This study introduces RobMedNAS, an innovative neural architecture search method designed to elevate the robustness of U-Net architectures in medical image synthesis against adversarial attacks. The significant achievement of RobMedNAS lies in its ability to autonomously identify U-Net architectures that maintain high accuracy on clean data while demonstrating superior resilience to adversarial perturbations. This balance is paramount in medical imaging, where the integrity and reliability of synthesized images are crucial for diagnostic accuracy and patient care.

The novelty of our work resides in the application of Bayesian optimization to the complex challenge of enhancing adversarial robustness in medical imaging models, presenting a novel path toward securing AI-driven medical applications. By successfully navigating the extensive landscape of potential U-Net configurations, RobMedNAS sets a new standard for the development of robust neural networks in healthcare technologies.

As we look to the future, our focus will shift towards refining and expanding the capabilities of RobMedNAS. Immediate plans include exploring its applicability to more complex and diverse medical imaging tasks beyond the synthesis of CT images from MRI inputs. This future work aims to further validate the robustness and efficiency of the architectures identified by RobMedNAS across a broader range of clinical scenarios and imaging modalities.

## Data Availability

The data cannot be made publicly available upon publication due to legal restrictions preventing unrestricted public distribution. The data that support the findings of this study are available upon reasonable request from the authors.

## Supplementary materials

In TPE, we impose a uniform distribution on each hyperparameter ${w}_{i}$ as the prior $p\left({w}_{i}\right),$ for $i=1,2,\,\ldots ,\,C,$ where $C$ is the total number of hyperparameters to be optimized. Then at the $\left(k+1\right)$-th iteration, we collect all historical explored architectures $x$ and their corresponding robustness metrics $y,$ denoted by ${D}^{k}=\left\{\left({x}^{1},{y}^{1}\right),\ldots ,\left({x}^{k},{y}^{k}\right)\right\}.$ Note that each architecture can be represented as a vector of hyperparameters, i.e., ${x}^{k}=\left({w}_{1}^{k},\,\ldots ,\,{w}_{C}^{k}\right).$ Thus, for the $i$-th hyperparameter ${w}_{i},$ we can obtain $k$ samples $\left\{\left({w}_{i}^{1},{y}^{1}\right),\,\ldots ,\,\left({w}_{i}^{k},{y}^{k}\right)\right\}$ to estimate the posterior distribution $p\left({w}_{i}| y\right),$ which are explained in [22] in detail. Given the prior distribution $p\left({w}_{i}\right)$ and the estimated posterior distribution $p\left({w}_{i}| y\right),$ we use the criterion of Expected Improvement (EI) [39] to identify the most promising value for ${w}_{i}.$ Finally, the collection of all hyperparameters with the most promising values forms the architecture ${x}^{k+1},$ and is trained and evaluated on the test data in the subdataset. After $N$ iterations, the architecture with the best robustness metric $y$ is output.
